# In vivo surveillance and elimination of teratoma‐forming human embryonic stem cells with monoclonal antibody 2448 targeting annexin A2

**DOI:** 10.1002/bit.27135

**Published:** 2019-08-30

**Authors:** Heng Liang Tan, Bao Zhu Tan, Winfred Xi Tai Goh, Simeon Cua, Andre Choo

**Affiliations:** ^1^ Bioprocessing Technology Institute, Agency for Science, Technology and Research (A*STAR) Biopolis Singapore

**Keywords:** antibody‐dependent cell‐mediated cytotoxicity, antibody‐drug conjugate, cancer, human embryonic stem cells, monoclonal antibody, pluripotent, teratoma

## Abstract

This study describes the use of a previously reported chimerised monoclonal antibody (mAb), ch2448, to kill human embryonic stem cells (hESCs) in vivo and prevent or delay the formation of teratomas. ch2448 was raised against hESCs and was previously shown to effectively kill ovarian and breast cancer cells in vitro and in vivo. The antigen target was subsequently found to be Annexin A2, an oncofetal antigen expressed on both embryonic cells and cancer cells. Against cancer cells, ch2448 binds and kills via antibody‐dependent cell‐mediated cytotoxicity (ADCC) and/or antibody‐drug conjugate (ADC) routes. Here, we investigate if the use of ch2448 can be extended to hESC. ch2448 was found to bind specifically to undifferentiated hESC but not differentiated progenitors. Similar to previous study using cancer cells, ch2448 kills hESC in vivo either indirectly by eliciting ADCC or directly as an ADC. The treatment with ch2448 post‐transplantation eliminated the in vivo circulating undifferentiated cells and prevented or delayed the formation of teratomas. This surveillance role of ch2448 adds an additional layer of safeguard to enhance the safety and efficacious use of pluripotent stem cell‐derived products in regenerative medicine. Thereby, translating the use of ch2448 in the treatment of cancers to a proof of concept study in hESC (or pluripotent stem cell [PSC]), we show that mAbs can also be used to eliminate teratoma forming cells in vivo during PSC‐derived cell therapies. We propose to use this strategy to complement existing methods to eliminate teratoma‐forming cells in vitro. Residual undifferentiated cells may escape in vitro removal methods and be introduced into patients together with the differentiated cells.

## Graphical abstract

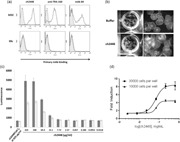

## 1 INTRODUCTION

Pluripotent stem cells (PSCs), which includes human embryonic stem cells (hESCs) and induced pluripotent stem cells (iPSCs), possess the dual properties of proliferating indefinitely and differentiating into cell types representing the three germ layers (Bongso & Richards, 2004; Gerecht‐Nir & Itskovitz‐Eldor, 2004; Takahashi et al., 2007). Studies have shown that PSCs can be differentiated in vitro to lineage‐specific cell types, such as neural‐like cells, insulin‐producing cells and cardiomyocytes with the goal of using these in regenerative medicine. Hence PSCs are a valuable source in transplantation to replace cells that have been damaged in the course of infections, diseases such as chronic heart disease, diabetes, and Parkinson's disease or due to congenital abnormalities (Angelos & Kaufman, 2015; Assady et al., 2001; Bongso & Richards, 2004; D'Amour et al., 2006; Efrat, 2002; Gerecht‐Nir & Itskovitz‐Eldor, 2004; He, 2003; Narazaki et al., 2008; Pankratz et al., 2007; Reubinoff et al., 2001; Schulz et al., 2003; Snir et al., 2003; Stewart, Stojkovic, & Lako, 2006; Thomson et al., 1998).

However, one of the safety concerns associated with the use of PSCs in cell‐based therapies is the presence of residual undifferentiated PSCs in the differentiated cell product, which potentially can form teratomas in patients (Choo et al., 2008; Schriebl et al., 2012; Tan, Fong, Lee, Yap, & Choo, 2009). Hentze et al. (2007) demonstrated that 245 undifferentiated hESCs were sufficient to form teratomas in severe combined immunodeficient (SCID) mice. Fujikawa et al. (2005) also showed that ES cell‐derived insulin expressing cells, post‐transplantation into SCID mice, formed teratomas in vivo resulting in the failure of treatment for Type I diabetes. In a prominent case study, a group of Israeli researchers reported that a boy who was suffering from ataxia telangiectasia, had received fetal neural stem cell transplants and developed tumors in his brain and spinal cord 4 years after treatment (Alper, 2009; Amariglio et al., 2009). This case study highlights the risk of teratoma or tumor formation not just from pluripotent stem cells but also from other sources of stem cells (including fetal neural stem cells) and will be a major stumbling block for cell‐based therapies.

Hentze, Graichen, and Colman (2007) summarized various methods to eliminate unwanted cells and enrich for the desired cell types in PSC‐cell therapies. These methods ranged from using genetically engineered PSCs, introducing miRNA switches, and purification based on physical methods to mitotically inactivate the cell therapy products. Many studies were also carried out to ensure the safety of PSC‐derived cell therapy. Pretreatment of teratoma‐forming cells with ceramide analogues, nanoparticles (mica fine particles), small molecule inhibitors and cardiac glycosides had been shown to prevent the formation of teratomas in vivo (Bieberich, Silva, Wang, Krishnamurthy, & Condie, 2004; Jeong, Cho, Lee, & Cha, 2017; Lee et al., 2013; Lin et al., 2017; Mohseni, Hamidieh, Verdi, & Shoae‐Hassani, 2014; Parr et al., 2016).

Antibodies specific to hESCs have been used to remove residual hESCs from mixed cell populations (Schriebl et al., 2012). Our group has also reported two cytotoxic monoclonal antibodies (mAbs) that kill PSCs via oncosis (Choo et al., 2008; Tan et al., 2009; Zheng, Tan, Matsudaira, & Choo, 2017). Recently, Sougawa et al. (2018) demonstrated that the antibody‐drug conjugate (ADC), Brentuximab vedotin, was able to kill PSCs. All these methods reported, involves the pretreatment of the teratoma‐forming cells in vitro before implantation.

In this paper, we utilized a previously reported mAb, ch2448, and showed that the mAb is able to kill PSCs. The mAb was raised against hESCs (HES‐3) in Balb/C mice and was chimerised with a human IgG_1_ backbone (Cua et al., 2018). Adsorption parameters for the antibody were previously determined by Schriebl et al. (2012). There, they observed that the maximum binding capacity (*q*_m_) of mAb 2448 to hESC is 138 ± 4.7 × 10^−18^ mol/hESC, which corresponds to 8.3 × 10^7^ molecules/hESC. Cua et al. (2018) had previously carried out a detailed characterization of this chimerised antibody. Besides binding to hESCs, ch2448 was found to bind to various cancer cells and the antigen target was identified as Annexin A2. ch2448 was also able to elicit antibody‐dependent cell‐mediated cytotoxicity (ADCC) and prevent cancer‐tumor formation in xenograft mouse models. Here, we demonstrate that ch2448 is able to elicit 2 types of mechanisms of action (MOAs) to kill hESCs. As a naked antibody, ch2448 is able to elicit ADCC. Additionally, its ability to be internalized into hESCs after binding creates an opportunity for ADC development. In both cases, we show that ch2448 is able to kill hESCs in vivo and prevent or delay the formation of teratomas.

## 2 MATERIALS AND METHODS

### 2.1 hESC culture

Human embryonic stem cell line, HES‐3 (46 X, X) was obtained from ES Cell International (ESI, Singapore). The cells were cultured at 37°C in a humidified atmosphere with 5% CO_2_, on matrigel‐coated culture dishes (Falcon) with daily replacement of mTeSR^TM^1 media (Stemcell Technologies). Cells were passaged by mechanically cutting with cell cutters (Invitrogen) into small square cell sheets, scraping from the culture dish using cell scrapers and transferring to freshly Matrigel‐coated culture dishes. Culture dishes were preincubated with matrigel (Corning) at 4°C overnight.

### 2.2 EBs formation

To induce differentiation in vitro, hESC were mechanically cut and harvested as clumps and cultured as embryoid bodies (EBs) for 7 days in EB media (80% KO‐DMEM, 20% fetal bovine serum [FBS], 25 U/ml penicillin, 25 µg/ml streptomycin, 2 mM l‐glutamine, 0.1 mM non‐essential amino acid (NEAA), and 0.1 mM 2‐mercaptoethanol) on a nonadherent six‐well plate (Nunc). Subsequently, the EBs were dissociated with trypsin (Invitrogen), plated on a gelatinized six‐well plate and passaged every 7 days for a fortnight. EB media was replaced once every 2 days.

### 2.3 Flow cytometry analysis

Cells were dissociated with trypsin and collected via low‐speed centrifugation. Each sample containing approximately 1 × 10^5^ single cells were resuspended in 1% bovine serum albumin/phosphate buffer saline (BSA/PBS) and incubated with 5 µg of ch2448, ch2448F(ab′)_2_, TRA‐160 (Millipore), or mAb 84 for 45 min on ice. Samples were washed with 1% BSA/PBS before incubating with fluorescein isothiocyanate (FITC)‐labelled goat anti‐human kappa light chain mAbs (Sigma Aldrich) or FITC‐labelled goat anti‐mouse immunoglobulin (Ig) polyclonal (Dako) for 15 min at room temperature. Samples were then washed twice with 1% BSA/PBS before reading on a FACScan (Becton Dickson FACSCalibur).

### 2.4 ADCC

ADCC activity of ch2448 was measured using the Promega ADCC Reporter Bioassay (Promega). Briefly, hESCs were harvested using dispase (Stem Cell Technologies) and washed in PBS. Cells were added to a 96‐well black culture plate (Corning) at 30,000 cells per well in mTeSR medium (Stem Cell Technologies). Dilutions of ch2448 were added at 25 μl per well. Engineered effector Jurkat cells, expressing the human FcγIIIA receptor coupled to a downstream gene reporter readout of the nuclear factor of the activated T‐cells (NFAT) ADCC signalling pathway, were thawed and added at approximately 75,000 cells per well. Post 6 hr of incubation, the culture plate was removed and luciferin solution added. Luminescence readout was done using a plate reader (TECAN M2000).

### 2.5 F(ab′)2 generation

The full‐length ch2448 was treated with IdeS protease (Genovis; FabRICATOR) at 1U per 1 μg of antibody at 37°C for 1 hr. The histadine‐tagged IdES was removed with a Ni‐NTA spin column (Qiagen).

### 2.6 Sodium dodecyl sulfate polyacrylamide gel electrophoresis (SDS‐PAGE) gel

The samples were boiled at 95°C after adding 5× sample loading dye and subjected to SDS‐PAGE using 4–12% gradient NuPAGE Bis‐Tris gel (#NP0335 Box) with 1× 3‐(*N*‐morpholino)propanesulfonic acid buffer (#NP001; Life Technologies). The proteins were separated at 110 V for 1 hr and subsequently stained with coomassie blue.

### 2.7 Biotinylation of mAbs

The mAb was biotinylated using the EZ‐Link Sulfo‐NHS‐Biotin kit (Thermo Fisher Scientific). Briefly, 50 µl of Biotin Reagent was added to 1 ml of ch2448 (2 mg/ml in PBS) and incubated at room temperature for 30 min. Nonreacted biotin was removed by dialysis.

### 2.8 Internalization studies

Biotinylated ch2448 were incubated with equimolar of pHRodo Red Avidin (Thermo Fisher Scientific, #P35362) in the dark and on ice for 5 min. The conjugated mAb (20 μg) was added to the hESC culture and real‐time visualization of the internalization was carried out by video capture on a DeltaVision (GE Healthcare Life Sciences). For the counter‐staining with Phallodin, after the internalization of ch2448 conjugated to pHRodo, the hESC culture was washed twice with cold PBS and subsequently fixed with 4% paraformaldehyde/PBS for 15 min. The cells were washed twice with cold PBS and permeabilized with 0.5% Triton‐X/PBS for 10 min. The washing was repeated and cells were blocked with 10% FBS/PBS for 10 min. The cells were washed twice with PBS and incubated with Phallodin conjugated to AlexaFluor 488 for 30 min in the dark. Excess dyes were washed off with PBS and 500 µl 1% BSA/PBS was added to each well before imaging.

### 2.9 Treatment of hESC with mAb and ADC

hESCs were seeded in a 24‐well plate, at a density of approximately 1 × 10^5^ cells/well. Antibody and ADCs were spiked into the hESC cultures post 24 hr seeding. The ADC cocktails were prepared by incubating ch2448F(ab′)_2_ with various secondary drugs, DM1 (Moradec), monomethyl auristatin E (MMAE; Moradec) at a 1:1 molar ratio for 15 min.

### 2.10 CellTiter‐Glo (CTG) luminescent assay

Metabolically active cells were measured based on the presence of ATP, using the CellTiter‐Glo Luminescent Cell Viability Assay Kit (Promega). An equal volume of CTG substrate (to media volume) was added to each 24‐well containing hESCs treated with the mAb and incubated for 15 min in the dark at RT on a shaker. Two hundred microliters of the lysed cells/substrate mix was pipetted to 96‐well plates (black, clear flat bottom). Luminescence was measured using a TECAN M2000. Experiments were carried out twice (two biological repeats).

### 2.11 Animal model

To carry out the teratoma formation of hESCs in vivo, cells were harvested with PBS and treated accordingly. hESCs were incubated with the ch2448, ch2448F(ab′)_2_, or ch2448F(ab′)_2_‐MMAE on ice for 45 min. The single‐cell suspension of hESCs (4 × 10^6^ cells per animal in a 30 μl PBS + 15 μl Matrigel mixture) was injected directly into the quadriceps of the right hind leg of a male SCID mouse. For the IP administration of the ADC, 35 μg of ch2448F(ab′)_2_‐MMAE was injected intraperitoneally on the day of hESC injection and the following dose, 24 hr later. Female NOD SCID mice (4–6‐week old) were purchased from InVivos. Animal experiments were performed in accordance with NIH and NACLAR guidelines (National University of Singapore IRB protocol 05–020, Biopolis IACUC approval 151006). Tumor formation was monitored visually using a simple grading system that was confirmed by caliper measurements: Grade 0, no visible teratoma (6.32‐mm average maximal hind leg diameter); Grade 1, teratoma just detectable (10.55‐mm average); Grade 2,  teratoma obvious (13.2‐mm average); and Grade 3, teratoma impedes locomotion (14.52‐mm average).

### 2.12 Statistical analysis

Analysis of variance was carried out using PRISM software.

## 3 RESULTS

### 3.1 ch2448 binds specifically to hESC and exhibits ADCC in vitro

**Figure 1:**
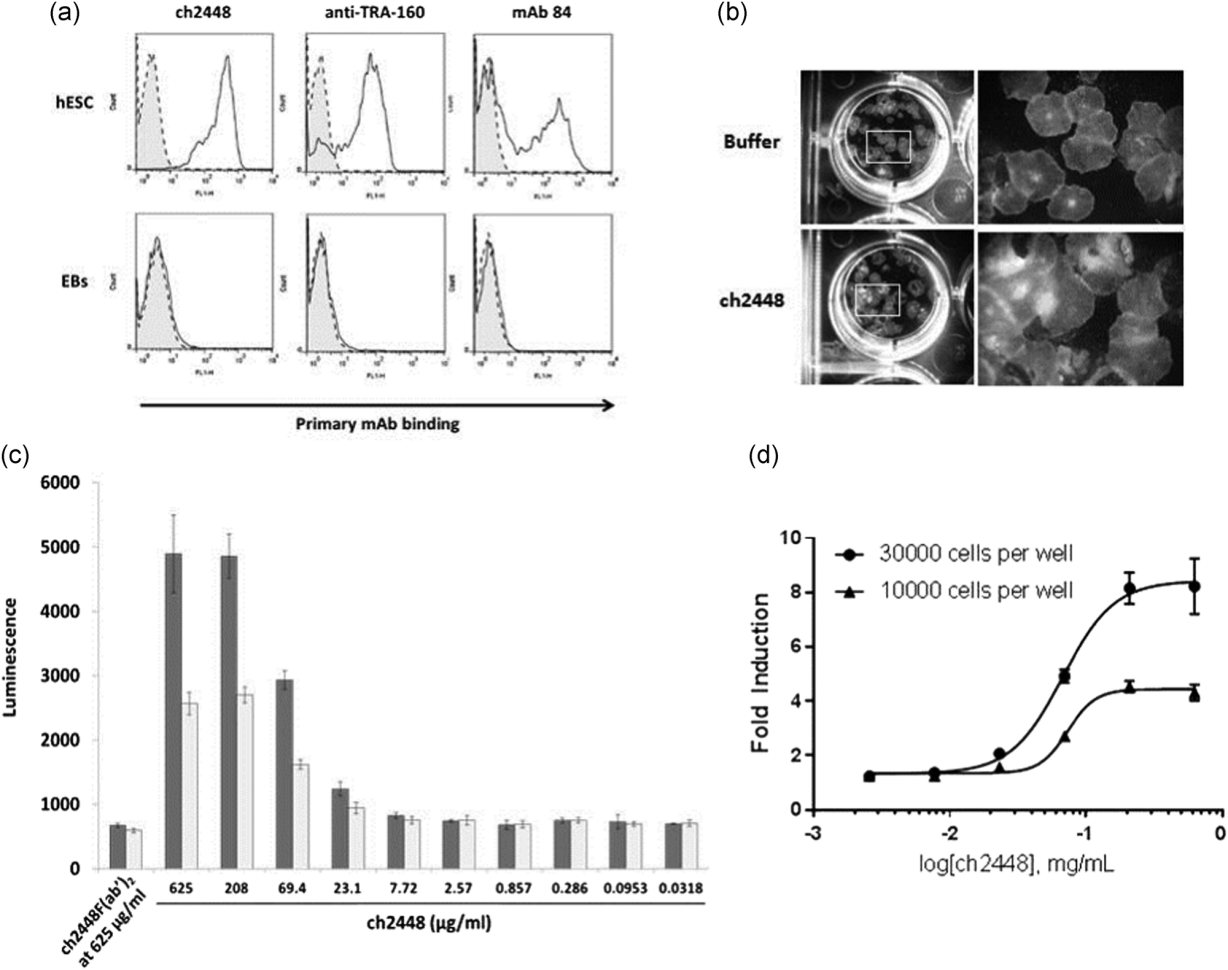
The antibody ch2448 binds to undifferentiated hESCs and elicits ADCC. (a) ch2448 binds to undifferentiated hESCs but not to 22‐day EBs. Shaded, dashed‐line histogram represents negative control. Bolded‐line histogram represents primary mAb binding. The undifferentiated hESC expressed the pluripotent markers, TRA‐160, and podocalyxin. The expression of these markers was lost in differentiated EBs. Detection of TRA‐160 and podocalyxin was carried out using commercially anti‐TRA‐160 and in‐house mAb 84 as primary antibodies, respectively. (b) ch2448 was incubated with hESC (20 μg/ml) over 72 hr. Buffer at an equivalent volume was added into another well of hESCs as control. In both wells, the proliferation of hESC was not affected. (c) ADCC activity of ch2448 on hESC. Single‐cell suspensions of hESCs were incubated with different concentrations of full‐length ch2448 and a reporter effector cell line for ADCC activity. A dose‐dependent increase in ADCC activity was observed. The ADCC assay was carried out by incubating the mAb with 30,000 cells and 10,000 cells (■ and □, respectively). (d) Induction of ADCC was measured as a fold increase against control wells containing ch2448F(ab′)_2_ at the highest concentration tested of ch2448. Values represent means ± standard deviation of biological triplicates. ADCC, antibody‐dependent cell‐mediated cytotoxicity; EB, embryoid body; hESC, human embryonic stem cell; mAb, monoclonal antibody

ch2488 was previously shown to bind to cancer and not to normal cells Cua et al. (2018). The binding of ch2448 to the immunogen hESC (HES‐3) was assessed via flow cytometry. Figure 1a shows that the mAb binds specifically to HES‐3 but not to differentiated embryoid bodies (EBs; left panel). The differentiated status of the EBs were also confirmed by the absence of pluripotent markers, TRA‐1–60 and podocalyxin compared with hESC (center and right panel). When ch2448 was spiked into hESC culture and incubated for 3 days, the cells continued to proliferate, similar to the buffer control (Figure 1b). Hence, as a naked mAb, ch2448 does not affect the proliferation of hESCs in vitro.

Cua et al. (2018) demonstrated that ch2448 was able to engage effector cells and elicit ADCC activity on target cancer cells. We proceeded to determine if ch2448 was able to elicit ADCC on hESCs. Increasing concentrations of ch2448 were incubated with hESCs in vitro, and the ADCC activity was measured via a gene reporter assay using engineered Jurkat cells that expressed the human Fc‐gamma IIIA receptor coupled to an ADCC NFAT signalling pathway as the readout. As a negative control, F(ab′)_2_ fragments of ch2448, termed ch2448F(ab′)_2_, were generated to eliminate the Fc‐mediated ADCC (Cua et al., 2018). The successful cleavage and generation of the F(ab′)_2_ fragments were confirmed on a SDS‐PAGE gel and binding of the fragments to hESCs was retained (Figure S1).

From Figure 1c, the negative control, ch2448F(ab′)_2_ (at the highest concentration of ch2448 tested), does not exhibit ADCC. The ADCC assay result also shows that ch2448 does exhibit ADCC in a dose‐dependent manner with about a maximum of eight‐fold induction when incubated with 30,000 hESCs. The induction of ADCC was measured as a fold increase against the negative control, ch2448F(ab′)_2_ at the highest concentration tested of ch2448 (Figure 1d). The data thus shows that the full‐length ch2448, a human IgG_1_, exhibits ADCC on hESCs in vitro and could possibly eliminate teratoma formation in vivo via Fc‐Fc receptor engagement with effector cells in mice.

### 3.2 ch2448 internalizes into undifferentiated hESCs

**Figure 2:**
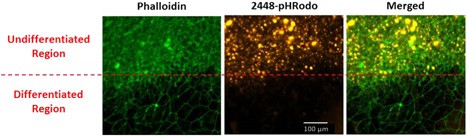
Internalisation of ch2448 occurs specifically in undifferentiated hESCs. ch2448 was incubated with live hESC culture and actin stained with Phallodin. Internalization of ch2448 (orange) was observed in the undifferentiated regions as characterized by the dense actin network (green). No internalization of ch2448 was observed in the differentiated regions (sparse actin network). hESC, human embryonic stem cell [Color figure can be viewed at wileyonlinelibrary.com]

Besides eliciting ADCC, ch2448 was found to exhibit a second MOA whereby it is internalized by the hESCs. To demonstrate this, the mAb was conjugated to pHRodo, a dye which fluoresces maximally at a low pH environment (e.g. in the endosome after internalization) and then incubated with live hESCs for 65 hr. The internalization of the mAb into the cells was captured real‐time and was found to occur as fast as 18 hr (see Supplementary Video; Figure S3 shows the localization of the mAb in the cells). In addition, ch2448 binds specifically to and is only internalized by undifferentiated cells and not differentiated cells. Here, ch2448 conjugated to pH‐Rodo, was first allowed to internalize into the live hESC culture which contained undifferentiated and differentiated regions. The cells were then fixed, permeabilized and stained with Phalloidin for actin. From Figure 2, regions of undifferentiated hESCs (characterized by the dense actin network) were stained positive for internalized ch2448. In contrast, the differentiated areas characterized by the sparse actin network were negative for internalized ch2448. This is consistent with the binding data (Figure 1a). Next, we proceeded to investigate if the antibody can be used as an ADC to kill hESCs.

### 3.3 Development of ch2448 as an ADC to undifferentiated hESCs in vitro

For an antibody to be developed as an ADC, apart from binding and internalization of the mAb into the target cells, it needs to be able to deliver a cytotoxic drug intracellularly, which consequently causes cell death (Modjtahedi, Ali, & Essapen, 2012; Pillay, Gan, & Scott, 2011; Scott, Wolchok, & Old, 2012). We have previously shown that the intact IgG, ch2448, can elicit ADCC. Hence, we tested if the ch2448F(ab′)_2_ can be used as an ADC in vitro. For the ADC assay, anti‐Fab secondary conjugated to two different cytotoxic agents that have been approved for clinical use were utilized. The first drug was emtansine (DM1) and the other was MMAE. Transtuzumab‐DM1 and Brentuximab Vedotin (anti‐CD30 conjugated to MMAE) are Food and Drug Administration (FDA)‐approved ADCs to treat breast cancers and Hodgkin's lymphoma, respectively (Pillay et al., 2011; Weiner, 2015).

**Figure 3:**
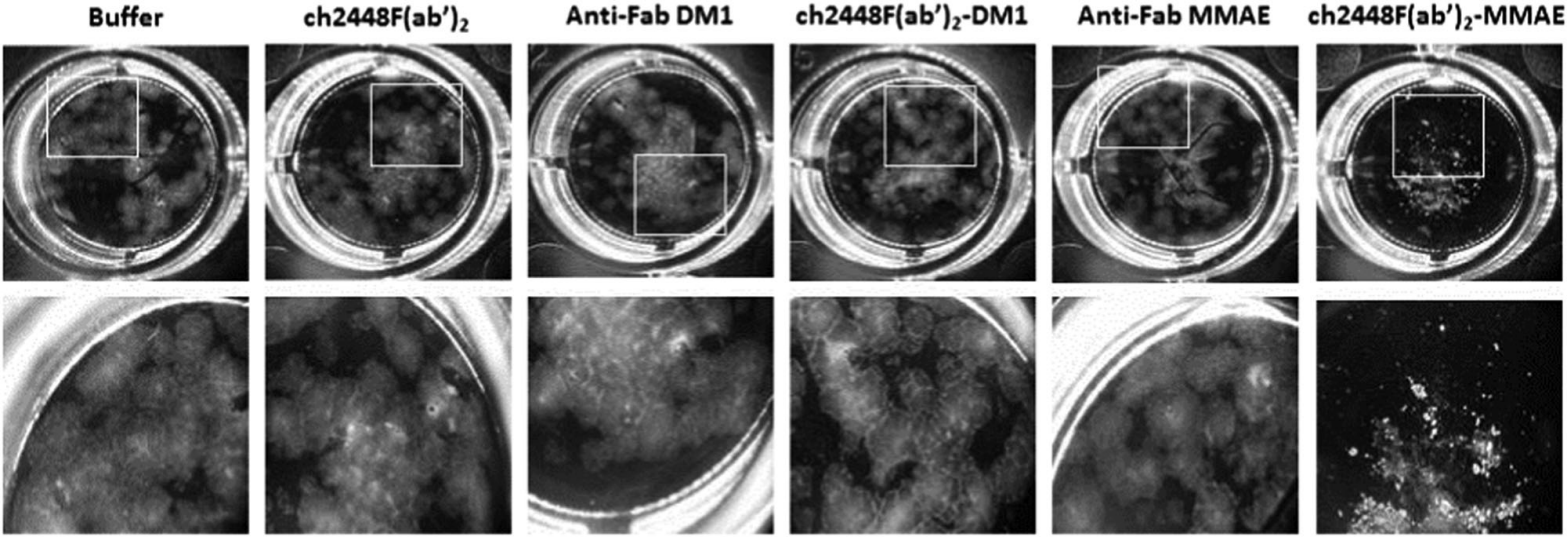
Effect of ch2448F(ab′)_2_ ADCs on hESCs (low dosage). ch2448F(ab′)_2_ was complexed with either anti‐Fab DM1 or anti‐Fab MMAE at a molar ratio of 1:1. The ch2448F(ab′)_2_‐ADCs were spiked into hESC culture, at a concentration of 1.5 μg/ml of ch2448F(ab′)_2_, and incubated for 5 days. Controls include buffer, ch2448F(ab′)_2_, anti‐Fab DM1, and anti‐Fab MMAE. ADC, antibody‐drug conjugate; DM1, emtansine; hESC, human embryonic stem cell; MMAE, monomethyl auristatin E

**Figure 4:**
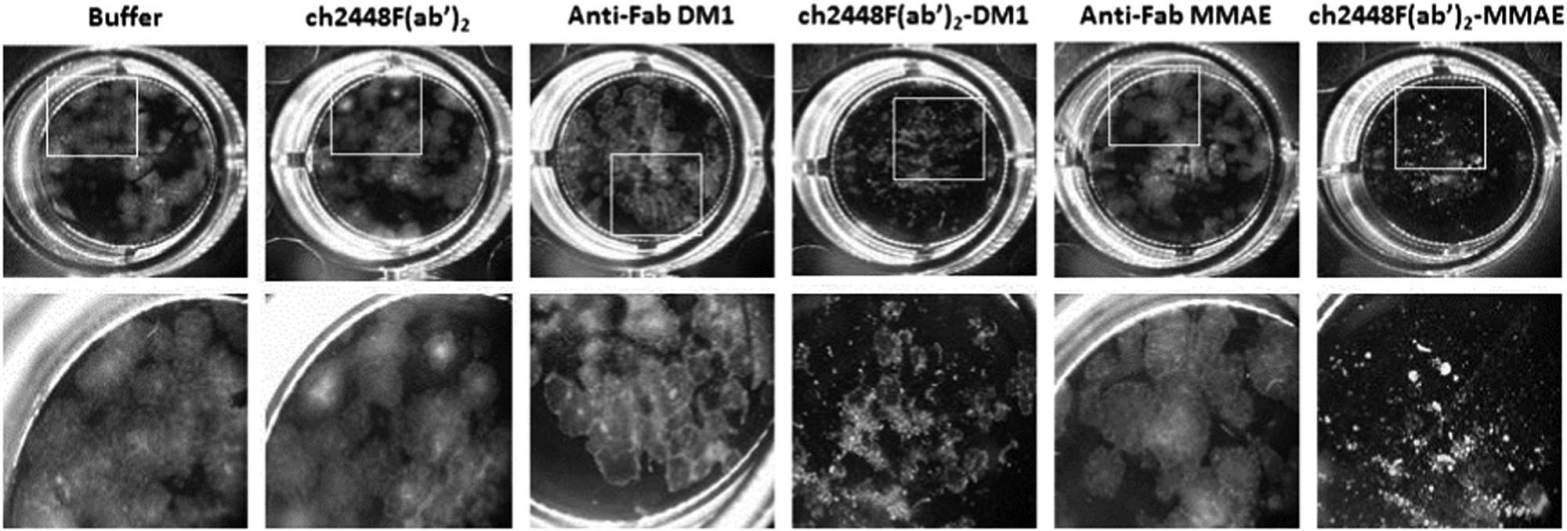
Effect of ch2448F(ab′)_2_ ADCs on hESCs (high dosage). ch2448F(ab′)_2_ was complexed with either anti‐Fab DM1 or anti‐Fab MMAE at a molar ratio of 1:1. The ch2448F(ab′)_2_‐ADCs were spiked into hESC culture, at a concentration of 3.0 μg/ml of ch2448F(ab′)_2_, and incubated for 5 days. Controls include buffer, ch2448F(ab′)_2_, anti‐Fab DM1 and anti‐Fab MMAE. ADC, antibody‐drug conjugate; hESC, human embryonic stem cell; MMAE, monomethyl auristatin E

**Figure 5:**
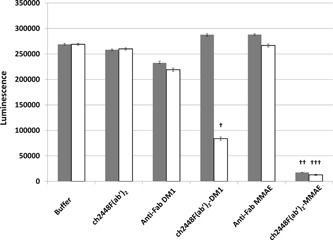
Total viable cell number post ch2448F(ab′)_2_‐ADCs treatment. ch2448F(ab′)_2_ was complexed with either anti‐Fab DM1 or anti‐Fab MMAE at a molar ratio of 1:1. The ch2448F(ab′)_2_ ADCs were spiked into hESC culture and incubated for 5 days. Controls include buffer, ch2448F(ab′)_2_, anti‐Fab DM1, and anti‐Fab MMAE. Total viable cell number was determined via CellTiter‐Glo® Luminescent Cell Viability Assay kit. Values are an average of six wells ± standard deviation. ■ represents the conditions at 1.5 μg/ml ch2448F(ab′)_2_, □ represents the conditions at 3.0 μg/ml ch2448F(ab′)_2_. ADC, antibody‐drug conjugate; DM1, emtansine; MMAE, monomethyl auristatin E. The cytotoxicities of the ADCs were significant when compared with the respective controls (Figure S2, ^†, ††, †††^ *p* < .01)

ch2448F(ab′)_2_ was first incubated with either of the two different anti‐Fab secondary (anti‐Fab DM1 and anti‐Fab MMAE) at a molar ratio of 1:1. Subsequently, the ADC complexes, ch2448F(ab′)_2_‐DM1 and ch2448F(ab′)_2_‐MMAE, were spiked into hESC cultures, seeded on the previous day. As controls, the cultures were spiked with either the buffer, ch2448F(ab′)_2_, or the two anti‐Fab secondary. The cultures were allowed to grow for an additional 5 days. After which, the cultures were visualized by microscopy (Figures 3 and 4) and total viable cell numbers assessed using the CellTiter‐Glo® Luminescent Cell Viability Assay kit (Figure 5). From Figures 3 and 4, the control cells formed colonies and proliferated to confluency. In contrast, cells treated with the ADCs, did not proliferate or form any colonies and cell debris was observed (Figure 4). From Figure 5, in the controls, the total viable cell numbers were high and comparable for cells treated with the buffer, ch2448F(ab′)_2_ and anti‐Fab MMAE. Slight cytotoxicity was however observed in the anti‐Fab DM1 condition. At 1.5 μg/ml, ch2448F(ab′)_2_, ch2448F(ab′)_2_‐DM1 showed no cytotoxicity, whereas treatment with ch2448F(ab′)_2_‐MMAE exhibited more than 90% cell death. Increasing the concentration of ch2448F(ab′)_2_ to 3.0 μg/ml resulted in increased cell cytotoxicity by ch2448F(ab′)_2_‐DM1 to approximately 60% compared with ch2448F(ab′)_2_‐MMAE where cell death was more than 90%. From these data, we concluded that ch2448F(ab′)_2_ conjugated to MMAE is a more potent ADC to kill undifferentiated hESC in vitro.

### 3.4 In vivo models for elimination and suppression of teratoma formation

To demonstrate the ability of ch2448 to eliminate teratoma formation through the different MOAs, namely; ADCC and ADC, several mice models were carried out. Teratoma formation was evaluated with a grading method as previously described by Choo et al. (2008) and Tan et al. (2009).

**Figure 6:**
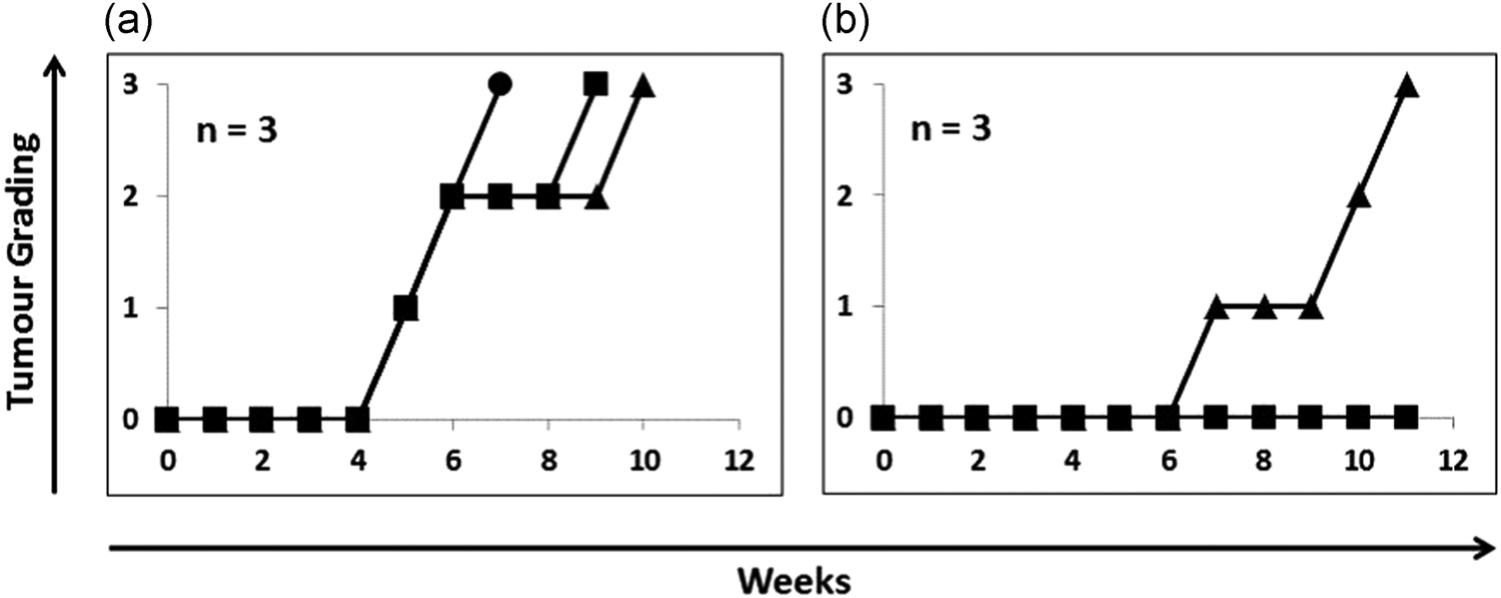
In vivo elimination and suppression of teratoma formation via ADCC. Single‐cell suspension of hESCs was pretreated with ch2448 in vitro and injected into the right hind leg muscles of SCID mice (*n* = 3). Killing via ADCC relied on the capacity of ch2448 to interact with the Fc receptors on the mouse effector cells in vivo. (a) Buffer control (b) hESC pretreated with ch2448. ADCC, antibody‐dependent cell‐mediated cytotoxicity; hESC, human embryonic stem cell; SCID, male severe combined immunodeficient

We first demonstrated the ability of ch2448 to kill hESC via ADCC in vivo. As previously shown, ch2448 does not kill hESC in vitro (Figure 1b). A single‐cell suspension of hESC was incubated with ch2448 in vitro and then injected intramuscularly into the right hind leg of SCID mice, relying on the capacity of ch2448 to engage mouse effector cells in vivo via Fc receptors (Cua et al., 2018). As control, hESC was pretreated with buffer only. In the buffer control (Figure 6a), teratomas started forming as early as Week 5 postinjection. In the ch2448‐treated group (Figure 6b), no teratoma was observed in two of the three mice. In one of the mice, teratoma formation started later, at Week 7. This first model shows that ch2448 is able to eliminate or delay the formation of teratomas in vivo via ADCC.

**Figure 7:**
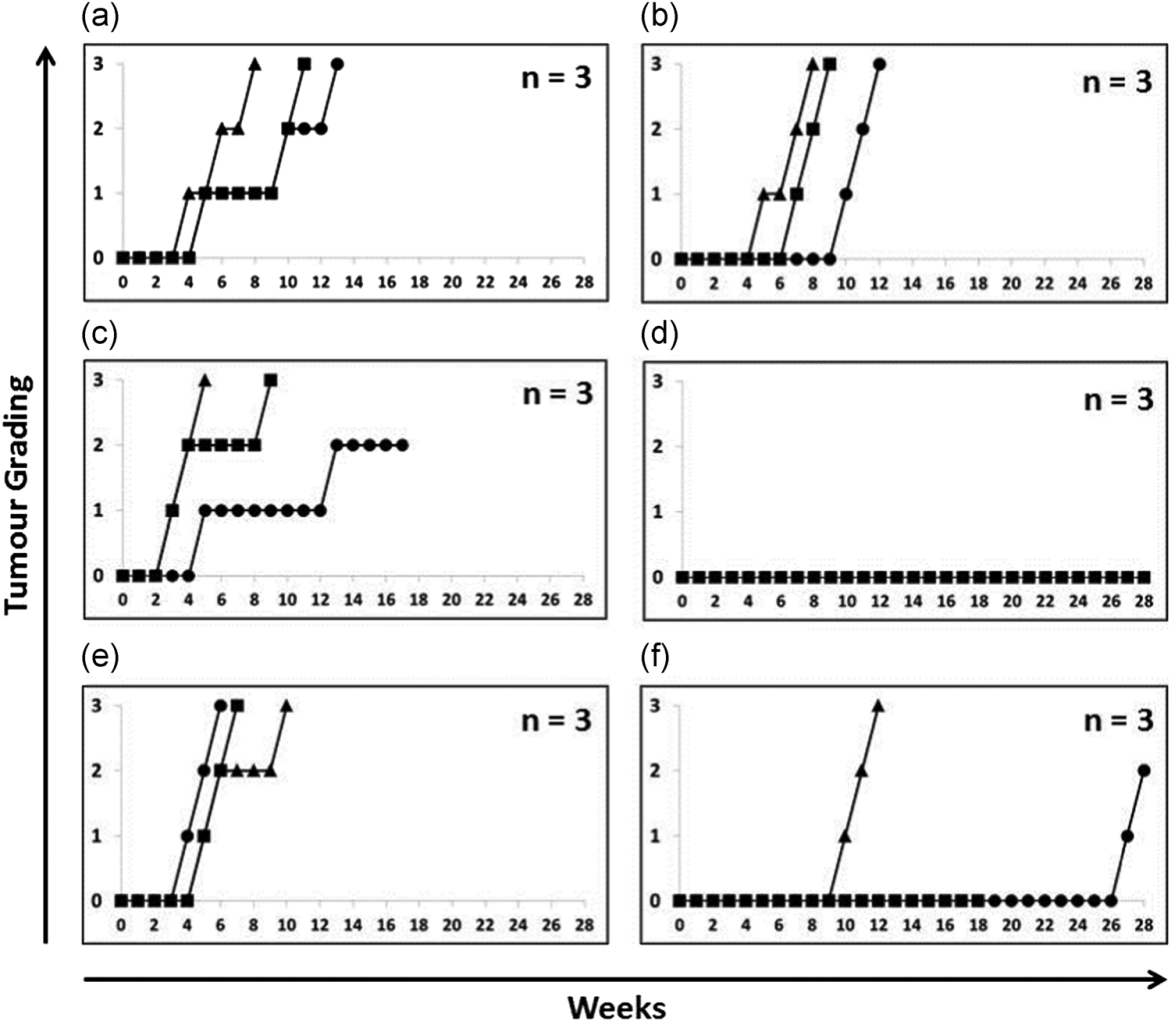
Elimination and suppression of teratoma formation via ADC. Single‐cell suspension of hESCs was pretreated with (a) buffer, (b) anti‐Fab MMAE, (c) ch2448F(ab′)_2_ and (d) ch2448F(ab′)_2_‐MMAE in vitro and injected intramuscularly into the right hind leg of SCID mice (*n* = 3). In another model, a single‐cell suspension of hESCs was first injected intramuscularly into the right hind leg of SCID mice (*n* = 3). (e) Buffer and (f) ch2448F(ab′)_2_‐MMAE administered intraperitoneally twice; on the day of cell injection and the following day. ADC, antibody‐drug conjugate; hESC, human embryonic stem cell; MMAE, monomethyl auristatin E; SCID, severe combined immunodeficient

Next, to demonstrate the ability of the mAb to kill hESC and prevent teratoma formation via ADC, a single‐cell suspension of hESCs was pretreated with the ADC, ch2448F(ab′)_2_‐MMAE, in vitro and injected intramuscularly into the right hind leg of SCID mice. The controls included pretreatment of hESC with buffer, anti‐Fab MMAE or ch2448F(ab′)_2_ fragments (Figure 7). In the buffer and anti‐Fab MMAE control (Figure 7a,b) teratoma formed as early as Week 4 in all mice. The ch2448F(ab′)_2_ did not elicit ADCC and did not prevent the formation of teratomas (Figure 7c). Interestingly, no teratoma formed in the ADC treated group even after 7 months (Figure 7d).

In our last animal model, untreated hESC was injected intramuscularly into the right hind leg of SCID mice and ch2448F(ab′)_2_‐MMAE was administered intraperitoneally; thus requiring the ADC to home onto the target cells through the vasculature. The ADC was administered twice, on the day of hESCs injection and the following day. In the buffer control, teratomas formed in all three mice as early as Week 4 (Figure 7e). For the mice that were administered with the ADC intraperitoneally, teratoma formation was either delayed or eliminated. Teratoma formation was significantly delayed in two mice (at Week 10 and Week 27). As for the third mouse in this treatment group, it was found dead after 18 weeks, however up until then, no teratoma formation was observed. This model is a proof of concept study that ADC can be administered as an in vivo surveillance for hESC to prevent teratoma formation.

## 4 DISCUSSION

In this study, we describe the MOAs of a previously reported antibody that was raised against hESC. There, Cua et al. characterized the antibody and found that the antigen target of ch2448 is Annexin A2. The mAb also elicited 2 MOAs to kill cancer cells, namely; ADCC and ADC. ch2448 also causes cancer tumour regression via ADCC in vivo via Fc‐Fc receptor engagement with effector cells in mice (Cua et al., 2018). Here, we show that ch2448 is also able to eliminate hESC via ADCC and ADC in vitro. Antibodies that exhibit ADCC and ADC properties are being used clinically to treat cancer patients (Scott, Allison, & Wolchok, 2012; Scott, Wolchok et al., 2012; Weiner, 2015). Hence, we translated the use of ch2448 in the treatment of cancers to a proof of concept study in hESC (PSC) and showed that ch2448 can eliminate PSC and prevent teratoma forming in vivo.

ch2448 by itself, does not kill hESC in vitro. However, as a chimerised mAb with a human IgG1 backbone, ch2448 is able to engage the Fc receptors on mice effector cells to elicit cell death. Here, we show that ch2448 is able to eliminate or delay teratoma formation via ADCC in vivo. The use of ch2448 to eliminate teratoma forming cells by ADCC can be further optimized by increasing the dose regime as shown by Cua et al. (2018) and by engineering a afucosylatyed ch2448, which can enhance ADCC by 20–25 folds.

The use of an ADC to eliminate stem cells has been previously reported (Sougawa et al., 2018). Sougawa et al. utilized the FDA approved ADC, Brentuximab vedontin, to eliminate undifferentiated CD30‐positive human iPSCs during cardiomyocyte differentiation and prevented teratoma formation. However, in their study, the elimination of undifferentiated cells was carried out in vitro before transplanting the treated cells into mice. Here, we demonstrated that pretreatment of undifferentiated hESC with ch2448F(ab′)_2_‐MMAE in vitro, is able to prevent the formation of teratomas in vivo. In addition, we showed that the ADC when administered at a site away from the cell transplant, is still able to home towards the hESCs and prevent or delay teratoma formation. Although one of the mice died midway through the experiment, the cause of death could not be determined. However, we do not attribute the cause of death to ch2448 as no adverse effect was observed in the mice by Cua et al. (2018). However, further optimization of the ADC is required to increase the efficacy of this mAb as an ADC drug. Direct conjugation of MMAE to ch2448, instead of using a secondary linked drug, will further enhance the stability of the ADC. Also, the drug dosage and regime can be increased.

In conclusion, ch2448 binds to undifferentiated hESCs and is able to elicit cell death via 2 MOAs in vivo and prevent or delay the formation of teratomas. However, the use of ch2448 must be further optimized and evaluated for specificity. Nonetheless, to our knowledge, this is the first report demonstrating the use of a mAb for in vivo surveillance and in vivo elimination of teratoma formation. We propose that this method can be used complementarily with existing methods to eliminate teratoma forming cells in vitro. Undifferentiated cells may escape the in vitro removal methods and be introduced into patients together with the differentiated cells post‐transplantation. mAbs that elicits ADCC and ADC serves as an additional safeguard to eliminate these “escaped” undifferentiated cells that are circulating in the body, thus enhancing the safety of PSCs‐derived cell therapies.

## CONFLICT OF INTERESTS

The authors declare that there are no conflict of interests.

## AUTHOR CONTRIBUTIONS

H. L. T. was responsible for the conception and design, collection and assembly of data, data analysis, interpretation and the manuscript writing. B. Z. T., W. X. T. G., and S. C. were responsible for the collection and assembly of data, data analysis, and interpretation. A. C. was responsible for the conception and design and final approval of the manuscript.

## Supporting information

Supporting information

Supporting information

## References

[bit27135-bib-0001] Alper, J. (2009). Geron gets green light for human trial of ES cell‐derived product. Nature Biotechnology, 27, 213–214. 10.1038/nbt0309-213a 19270655

[bit27135-bib-0002] Amariglio, N. , Hirshberg, A. , Scheithauer, B. W. , Cohen, Y. , Loewenthal, R. , Trakhtenbrot, L. , … Rechavi, G. (2009). Donor‐derived brain tumor following neural stem cell transplantation in an ataxia telangiectasia patient. PLOS Medicine, 6):e1000029 http://www.ncbi.nlm.nih.gov/pubmed/19226183 19226183 10.1371/journal.pmed.1000029 PMC2642879

[bit27135-bib-0003] Angelos, M. G. , & Kaufman, D. S. (2015). Pluripotent stem cell applications for regenerative medicine. Current Opinion in Organ Transplantation, 20, 1 http://www.ncbi.nlm.nih.gov/pubmed/26536430 26536430 10.1097/MOT.0000000000000244 PMC4635470

[bit27135-bib-0004] Assady, S. , Maor, G. , Amit, M. , Itskovitz‐Eldor, J. , Skorecki, K. L. , & Tzukerman, M. (2001). Insulin production by human embryonic stem cells. Diabetes, 50, 1691–1697. 10.2337/diabetes.50.8.1691. http://diabetes.diabetesjournals.org/content/50/8/1691.short%5Cn http://diabetes.diabetesjournals.org/cgi/ 11473026

[bit27135-bib-0005] Bieberich, E. , Silva, J. , Wang, G. , Krishnamurthy, K. , & Condie, B. G. (2004). Selective apoptosis of pluripotent mouse and human stem cells by novel ceramide analogues prevents teratoma formation and enriches for neural precursors in ES cell–derived neural transplants. Journal of Cell Biology, 167, 723–734. 15545317 10.1083/jcb.200405144 PMC2172580

[bit27135-bib-0006] Bongso, A. , & Richards, M. (2004). History and perspective of stem cell research. Best Practice & Research Clinical Obstetrics & Gynaecology, 18, 827–842. 15582541 10.1016/j.bpobgyn.2004.09.002

[bit27135-bib-0007] Choo, A. B. , Tan, H. L. , Ang, S. N. , Fong, W. J. , Chin, A. , Lo, J. , … Yap, M. (2008). Selection against undifferentiated human embryonic stem cells by a cytotoxic antibody recognizing podocalyxin‐like protein‐1. Stem Cells, 26, 1454–1463. http://doi.wiley.com/10.1634/stemcells.2007-0576 18356574 10.1634/stemcells.2007-0576

[bit27135-bib-0008] Cua, S. , Tan, H. L. , Fong, W. J. , Chin, A. , Lau, A. , Ding, V. , … Choo, A. (2018). Targeting of embryonic annexin A2 expressed on ovarian and breast cancer by the novel monoclonal antibody 2448. Oncotarget, 9, 13206–13221. http://www.ncbi.nlm.nih.gov/pubmed/29568351%0A http://www.pubmedcentral.nih.gov/articlerender.fcgi?artid=PMC5862572 29568351 10.18632/oncotarget.24152 PMC5862572

[bit27135-bib-0009] D'Amour, K. A. , Bang, A. G. , Eliazer, S. , Kelly, O. G. , Agulnick, A. D. , Smart, N. G. , … Baetge, E. E. (2006). Production of pancreatic hormone‐expressing endocrine cells from human embryonic stem cells. Nature Biotechnology, 24, 1392–1401. 10.1038/nbt1259 17053790

[bit27135-bib-0010] Efrat, S. (2002). Cell replacement therapy for type 1 diabetes. Trends in Molecular Medicine, 8, 334–340. 12114113 10.1016/s1471-4914(02)02365-1

[bit27135-bib-0011] Fujikawa, T. , Oh, S.‐H. , Pi, L. , Hatch, H. M. , Shupe, T. , & Petersen, B. E. (2005). Teratoma formation leads to failure of treatment for type i diabetes using embryonic stem cell‐derived insulin‐producing cells. The American Journal of Pathology, 166, 1781–1791. http://www.ncbi.nlm.nih.gov/pubmed/5920163 15920163 10.1016/S0002-9440(10)62488-1 PMC1602425

[bit27135-bib-0012] Gerecht‐Nir, S. , & Itskovitz‐Eldor, J. (2004). Cell therapy using human embryonic stem cells. Transplant Immunology, 12, 203–209. 15157914 10.1016/j.trim.2003.12.013

[bit27135-bib-0013] He, J.‐Q. , Ma, Y. , Lee, Y. , Thomson, J. A. , & Kamp, T. J. (2003). Human embryonic stem cells develop into multiple types of cardiac myocytes: Action potential characterization. Circulation Research, 93, 32–39. http://circres.ahajournals.org/cgi/doi/10.1161/01.RES.0000080317.92718.99 12791707 10.1161/01.RES.0000080317.92718.99

[bit27135-bib-0014] Hentze, H. , Graichen, R. , & Colman, A. (2007). Cell therapy and the safety of embryonic stem cell‐derived grafts. Trends in Biotechnology, 25, 24–32. 17084475 10.1016/j.tibtech.2006.10.010

[bit27135-bib-0015] Hentze, H. , Soong, P. L. , Wang, S. T. , Phillips, B. W. , Putti, T. C. , & Dunn, N. R. (2009). Teratoma formation by human embryonic stem cells: Evaluation of essential parameters for future safety studies. Stem Cell Research, 2, 198–210. 10.1016/j.scr.2009.02.002 19393593

[bit27135-bib-0016] Jeong, H. C. , Cho, S. J. , Lee, M. O. , & Cha, H. J. (2017). Technical approaches to induce selective cell death of pluripotent stem cells. Cellular and Molecular Life Science, 74, 2601–2611. 10.1007/s00018-017-2486-0 PMC11107638 28246701

[bit27135-bib-0017] Lee, M.‐O. , Moon, S. H. , Jeong, H.‐C. , Yi, J.‐Y. , Lee, T.‐H. , Shim, S. H. , … Cha, H.‐J. (2013). Inhibition of pluripotent stem cell‐derived teratoma formation by small molecules. Proceedings of the National Academy of Sciences, 110, E3281–E3290. http://www.pnas.org/cgi/doi/10.1073/pnas.1303669110 10.1073/pnas.1303669110 PMC3761568 23918355

[bit27135-bib-0018] Lin, Y.‐T. , Wang, C.‐K. , Yang, S.‐C. , Hsu, S.‐C. , Lin, H. , Chang, F.‐P. , … Lu, J. (2017). Elimination of undifferentiated human embryonic stem cells by cardiac glycosides. Scientific Reports, 7, 5289 http://www.nature.com/articles/s41598-017-05616-2 28706279 10.1038/s41598-017-05616-2 PMC5509667

[bit27135-bib-0019] Modjtahedi, H. , Ali, S. , & Essapen, S. (2012). Therapeutic application of monoclonal antibodies in cancer: Advances and challenges. British Medical Bulletin, 104, 41–59. 23118261 10.1093/bmb/lds032

[bit27135-bib-0020] Mohseni, R. , Hamidieh, A. A. , Verdi, J. , & Shoae‐Hassani, A. (2014). Safe transplantation of pluripotent stem cell by preventing teratoma formation. Journal of Stem Cell Research and Therapy, 04 https://www.omicsonline.org/open-access/safe-transplantation-of-pluripotent-stem-cell-by-preventing-teratoma-formation-2157-7633.1000212.php?aid=27424

[bit27135-bib-0021] Narazaki, G. , Uosaki, H. , Teranishi, M. , Okita, K. , Kim, B. , Matsuoka, S. , … Yamashita, J. K. (2008). Directed and systematic differentiation of cardiovascular cells from mouse induced pluripotent stem cells. Circulation, 118, 498–506. 18625891 10.1161/CIRCULATIONAHA.108.769562

[bit27135-bib-0022] Pankratz, M. T. , Li, X.‐J. , LaVaute, T. M. , Lyons, E. A. , Chen, X. , & Zhang, S.‐C. (2007). Directed neural differentiation of human embryonic stem cells via an obligated primitive anterior stage. Stem Cells, 25, 1511–1520. http://doi.wiley.com/10.1634/stemcells.2006-0707 17332508 10.1634/stemcells.2006-0707 PMC2743478

[bit27135-bib-0023] Parr, C. J. C. , Katayama, S. , Miki, K. , Kuang, Y. , Yoshida, Y. , Morizane, A. , … Saito, H. (2016). MicroRNA‐302 switch to identify and eliminate undifferentiated human pluripotent stem cells. Scientific Reports, 6, 1–14. 10.1038/srep32532 27608814 PMC5016789

[bit27135-bib-0024] Pillay, V. , Gan, H. K. , & Scott, A. M. (2011). Antibodies in oncology. New Biotechnology, 28, 518–529. 10.1016/j.nbt.2011.03.021 21473941

[bit27135-bib-0025] Reubinoff, B. E. , Itsykson, P. , Turetsky, T. , Pera, M. F. , Reinhartz, E. , Itzik, A. , & Ben‐Hur, T. (2001). Neural progenitors from human embryonic stem cells. Nature Biotechnology, 19, 1134–1140. 10.1038/nbt1201-1134 11731782

[bit27135-bib-0026] Schriebl, K. , Satianegara, G. , Hwang, A. , Tan, H. L. , Fong, W. J. , Yang, H. H. , … Choo, A. (2012). Selective removal of undifferentiated human embryonic stem cells using magnetic activated cell sorting followed by a cytotoxic antibody. Tissue Engineering. Part A, 18, 899–909. https://www.liebertpub.com/doi/10.1089/ten.tea.2011.0311 22092252 10.1089/ten.TEA.2011.0311

[bit27135-bib-0027] Schulz, T. C. , Palmarini, G. M. , Noggle, S. A. , Weiler, D. A. , Mitalipova, M. M. , & Condie, B. G. (2003). Directed neuronal differentiation of human embryonic stem cells. BMC Neuroscience, 4, 27. 14572319 10.1186/1471-2202-4-27 PMC272931

[bit27135-bib-0028] Scott, A. M. , Allison, J. P. , & Wolchok, J. D. (2012). Monoclonal antibodies in cancer therapy. Cancer immunity: a journal of the Academy of Cancer Immunology, 12, 14. 22896759 PMC3380347

[bit27135-bib-0029] Scott, A. M. , Wolchok, J. D. , & Old, L. J. (2012). Antibody therapy of cancer. Nature Reviews Cancer, 12, 278–287. http://www.nature.com/doifinder/10.1038/nrc3236 22437872 10.1038/nrc3236

[bit27135-bib-0030] Snir, M. , Kehat, I. , Gepstein, A. , Coleman, R. , Itskovitz‐Eldor, J. , Livne, E. , & Gepstein, L. (2003). Assessment of the ultrastructural and proliferative properties of human embryonic stem cell‐derived cardiomyocytes. American Journal of Physiology—Heart and Circulatory Physiology, 285, H2355–H2363. http://www.ncbi.nlm.nih.gov/pubmed/14613910 14613910 10.1152/ajpheart.00020.2003

[bit27135-bib-0031] Sougawa, N. , Miyagawa, S. , Fukushima, S. , Kawamura, A. , Yokoyama, J. , Ito, E. , … Sawa, Y. (2018). Immunologic targeting of CD30 eliminates tumourigenic human pluripotent stem cells, allowing safer clinical application of hiPSC‐based cell therapy. Scientific Reports, 8, 1–12. 10.1038/s41598-018-21923-8 29487310 PMC5829260

[bit27135-bib-0032] Stewart, R. , Stojkovic, M. , & Lako, M. (2006). Mechanisms of self‐renewal in human embryonic stem cells. European Journal of Cancer, 42, 1257–1272. 16630716 10.1016/j.ejca.2006.01.033

[bit27135-bib-0033] Takahashi, K. , Tanabe, K. , Ohnuki, M. , Narita, M. , Ichisaka, T. , Tomoda, K. , … Yamanaka, S. (2007). Induction of pluripotent stem cells from adult human fibroblasts by defined factors. Cell, 131, 861–872. https://linkinghub.elsevier.com/retrieve/pii/S0092867407014717 18035408 10.1016/j.cell.2007.11.019

[bit27135-bib-0034] Tan, H. L. , Fong, W. J. , Lee, E. H. , Yap, M. , & Choo, A. (2009). mAb 84, a cytotoxic antibody that kills undifferentiated human embryonic stem cells via oncosis. Stem Cells, 27, 1792–1801. 19544435 10.1002/stem.109

[bit27135-bib-0035] Thomson, J. A. , Itskovitz‐Eldor, J. , Shapiro, S. S. , Waknitz, M. A. , Swiergiel, J. J. , Marshall, V. S. , … Jones, J. M. (1998). Embryonic stem cell lines derived from human blastocysts. Science, 282, 1145–1147. http://www.ncbi.nlm.nih.gov/pubmed/9804556 9804556 10.1126/science.282.5391.1145

[bit27135-bib-0036] Weiner, G. J. (2015). Building better monoclonal antibody‐based therapeutics. Nature Reviews Cancer, 15, 361–370. http://www.nature.com/doifinder/10.1038/nrc3930 25998715 10.1038/nrc3930 PMC4491443

[bit27135-bib-0037] Zheng, J. Y. , Tan, H. L. , Matsudaira, P. T. , & Choo, A. (2017). Excess reactive oxygen species production mediates monoclonal antibody‐induced human embryonic stem cell death via oncosis. Cell Death and Differentiation, 24, 546–558. http://www.nature.com/doifinder/10.1038/cdd.2016.164 28106884 10.1038/cdd.2016.164 PMC5344215

