# Supplementary material for: In vivo surveillance and elimination of teratoma‐forming human embryonic stem cells with monoclonal antibody 2448 targeting annexin A2
Source: Biotechnol Bioeng. 2019 Aug 30;116(11):2996–3005. doi: 10.1002/bit.27135 (PMC6790577; doi:10.1002/bit.27135)
Supplement: Supplementary file 1 — Supporting information [file BIT-116-2996-s001.pdf]

(A)

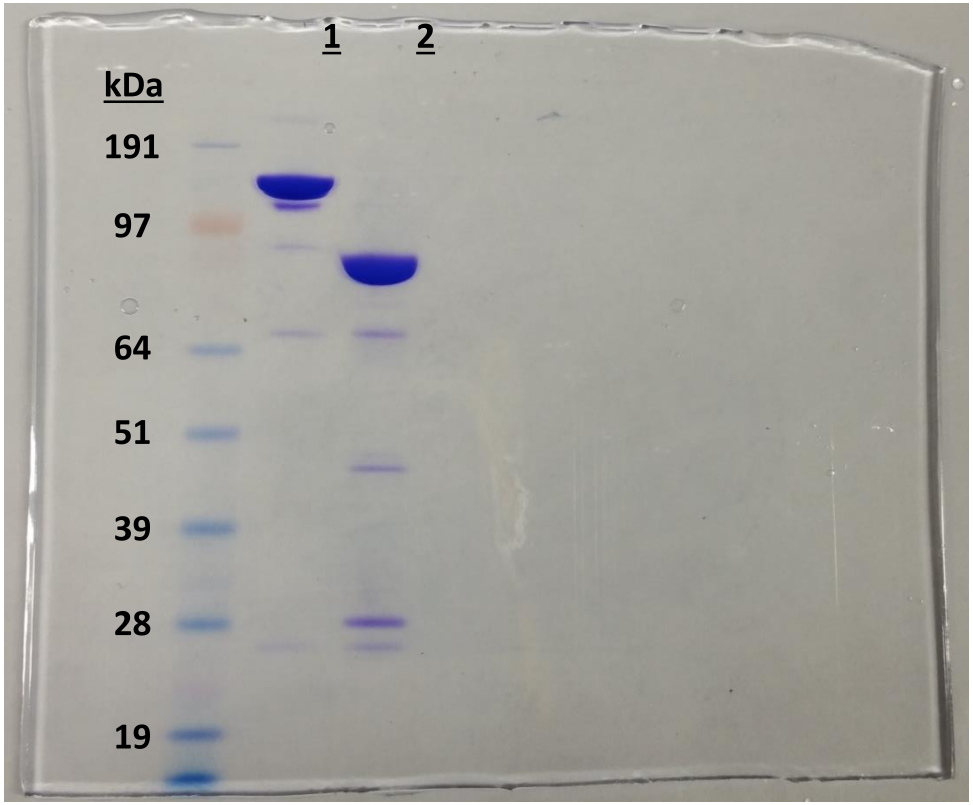

(B)

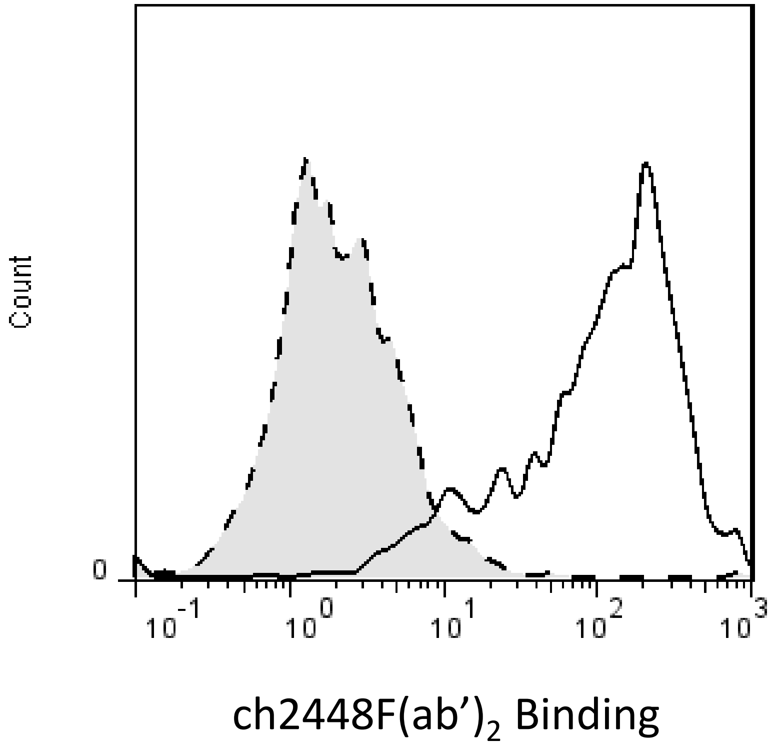

Figure Legend

Lane 1 : ch2448 IgG

Lane 2 : ch2448 F(ab')$_2$

Supplementary Figure 1. (A) Enzymatic digestion of IgG ch2448 (Lane 1) to F(ab')$_2$ (Lane 2). (B) Via flow cytometry, the F(ab')$_2$ fragments retained binding to undifferentiated hESCs. Shaded, dashed-line histogram represents negative control. Bolded-line histogram represents primary mAb binding.

Tukey's multiple comparisons test	Mean Diff.	95.00% CI of diff.	Significant?	Adjusted P Value
**1.5µg**				
Buffer vs. ch2448(Fab')2	10717	4917 to 16517	Yes	<0.0001
Buffer vs. Anti-Fab DM1	36480	30680 to 42280	Yes	<0.0001
Buffer vs. ch2448(Fab')2-DM1	-18743	-24543 to -12943	Yes	<0.0001
Buffer vs. Anti-Fab MMAE	-19145	-24945 to -13345	Yes	<0.0001
Buffer vs. ch2448(Fab')2-MMAE (††)	251511	245711 to 257311	Yes	<0.0001
ch2448(Fab')2 vs. Anti-Fab DM1	25762	19962 to 31562	Yes	<0.0001
ch2448(Fab')2 vs. ch2448(Fab')2-DM1	-29460	-35260 to -23660	Yes	<0.0001
ch2448(Fab')2 vs. Anti-Fab MMAE	-29862	-35662 to -24062	Yes	<0.0001
ch2448(Fab')2 vs. ch2448(Fab')2-MMAE (††)	240794	234994 to 246594	Yes	<0.0001
Anti-Fab DM1 vs. ch2448(Fab')2-DM1	-55223	-61023 to -49423	Yes	<0.0001
Anti-Fab DM1 vs. Anti-Fab MMAE	-55625	-61425 to -49825	Yes	<0.0001
Anti-Fab DM1 vs. ch2448(Fab')2-MMAE	215032	209232 to 220832	Yes	<0.0001
ch2448(Fab')2-DM1 vs. Anti-Fab MMAE	-402	-6202 to 5398	No	>0.9999
ch2448(Fab')2-DM1 vs. ch2448(Fab')2-MMAE	270254	264454 to 276054	Yes	<0.0001
Anti-Fab MMAE vs. ch2448(Fab')2-MMAE (††)	270656	264856 to 276456	Yes	<0.0001
**3.0µg**				
Buffer vs. ch2448(Fab')2	9061	3261 to 14861	Yes	0.0008
Buffer vs. Anti-Fab DM1	49987	44187 to 55787	Yes	<0.0001
Buffer vs. ch2448(Fab')2-DM1 (†)	185142	179342 to 190942	Yes	<0.0001
Buffer vs. Anti-Fab MMAE	2255	-3545 to 8055	No	0.8314
Buffer vs. ch2448(Fab')2-MMAE (†††)	256202	250402 to 262002	Yes	<0.0001
ch2448(Fab')2 vs. Anti-Fab DM1	40925	35125 to 46725	Yes	<0.0001
ch2448(Fab')2 vs. ch2448(Fab')2-DM1 (†)	176081	170281 to 181881	Yes	<0.0001
ch2448(Fab')2 vs. Anti-Fab MMAE	-6806	-12606 to -1006	Yes	0.015
ch2448(Fab')2 vs. ch2448(Fab')2-MMAE (†††)	247141	241341 to 252941	Yes	<0.0001
Anti-Fab DM1 vs. ch2448(Fab')2-DM1 (†)	135155	129355 to 140955	Yes	<0.0001
Anti-Fab DM1 vs. Anti-Fab MMAE	-47731	-53531 to -41931	Yes	<0.0001
Anti-Fab DM1 vs. ch2448(Fab')2-MMAE	206216	200416 to 212016	Yes	<0.0001
ch2448(Fab')2-DM1 vs. Anti-Fab MMAE	-182887	-188687 to -177087	Yes	<0.0001
ch2448(Fab')2-DM1 vs. ch2448(Fab')2-MMAE	71060	65260 to 76860	Yes	<0.0001
Anti-Fab MMAE vs. ch2448(Fab')2-MMAE (†††)	253947	248147 to 259747	Yes	<0.0001

Supplementary Figure 2. ANOVA was carried out across the various conditions using PRISM for both antibody dosages of 1.5 µg and 3.0 µg. The cytotoxicity of the ADCs were significant when compared to the respective controls (†, ††, †††; $p < 0.01$).

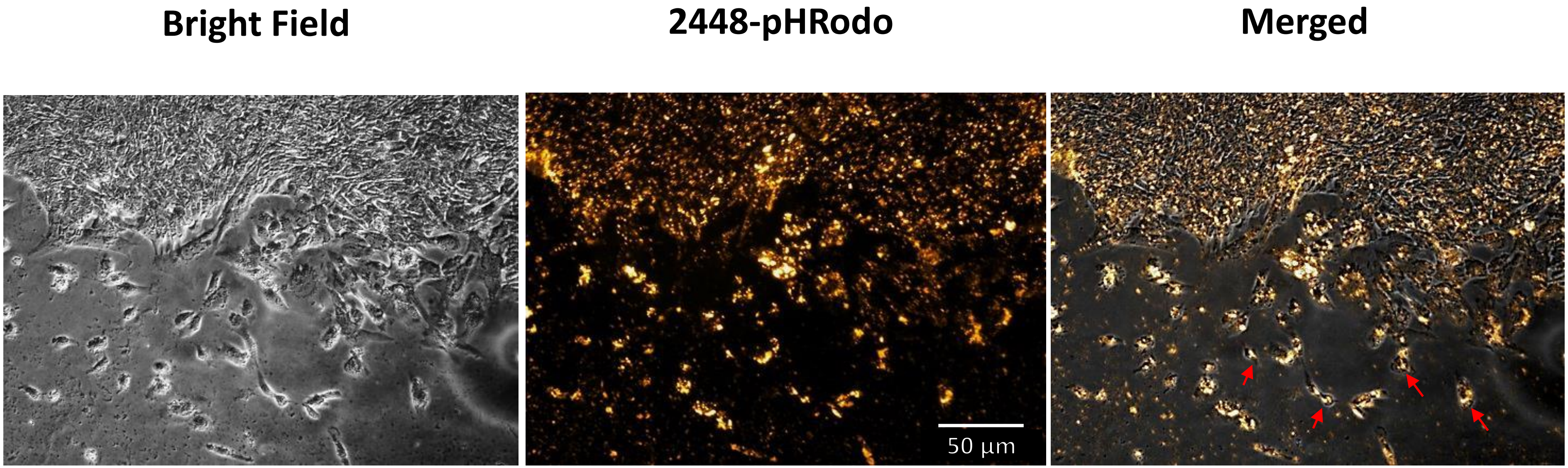

Supplementary Figure 3. ch2448 was conjugated to pHRodo dye which fluoresces maximally when it is internalized through the endosomes where the environment is acidic. When the cells were incubated with the mAbs, the antibody gets internalized and is observed to localize inside the cells. The red arrows indicate some cells with obvious internal localization of the mAb. The real time internalization of the antibody can be observed in the Supplementary Video.
